# Calciphylaxis: A Rare Manifestation of a Common Disease

**DOI:** 10.7759/cureus.84550

**Published:** 2025-05-21

**Authors:** Carlos Nancassa, Tetiana Baiherych, Adelaide Figueiredo

**Affiliations:** 1 Medicine, Unidade Local de Saúde da Lezíria, Hospital Distrital de Santarém, Santarém, PRT; 2 Internal Medicine, Hospital Distrital de Santarém, Santarém, PRT

**Keywords:** arteriolopathy, calcific skin, calciphylaxis, hypoparathyroidism, uremic

## Abstract

Calciphylaxis is a rare complication, typically observed in patients with end-stage chronic kidney disease (CKD), in the presence of risk factors such as secondary hyperparathyroidism, female sex, and diabetes mellitus, among others. It is characterized by painful cutaneous lesions resulting from calcification of arterioles, leading to vascular occlusion, tissue ischemia, and necrosis. We report the case of a 71-year-old woman with a history of CKD, Kidney Disease: Improving Global Outcomes G5 (KDIGO G5), secondary to diabetic nephropathy. As a relevant risk factor, she presented with secondary hyperparathyroidism associated with renal disease. She was admitted to the Emergency Department with erythematous and edematous plaques on both lower limbs, accompanied by severe pain. Laboratory evaluation revealed normocytic normochromic anemia, hyperphosphatemia, normal serum calcium levels, and elevated parathyroid hormone (PTH). The patient was hospitalized for etiological investigation. A skin biopsy obtained during hospitalization confirmed the diagnosis of calciphylaxis, revealing vascular calcifications and panniculitis. Treatment was initiated with opioids for pain control and topical heparinoid for the management of skin lesions. Following discharge, the patient remains under follow-up at the Nephrology clinic, with no recurrence of cutaneous lesions. This case underscores the importance of early diagnosis and a multidisciplinary approach in the management of calciphylaxis, a rare but potentially life-threatening condition associated with high mortality.

## Introduction

Calciphylaxis, also known as calcific uremic arteriolopathy (CUA), is a rare complication characterized by subcutaneous vascular calcification, most commonly observed in patients with advanced-stage chronic kidney disease (CKD) or undergoing hemodialysis [1-4]. However, there are reported cases of calciphylaxis in individuals with preserved renal function, a condition referred to as non-uremic calciphylaxis [2,4].

The term was originally coined by Hans Selye in 1962, based on experimental models [3,5,6]. Subsequently, the first documented clinical cases emerged in patients with CKD [5,6].

The incidence of CUA has increased over the past decade, with recent estimates suggesting a prevalence of approximately 5% among patients on hemodialysis, potentially related to growing clinical awareness and the availability of dedicated electronic registries [6,7]. Nevertheless, the overall prevalence of calciphylaxis remains unknown [4,7,8].

The etiology of calciphylaxis is multifactorial, with several risk factors implicated, including female sex, Caucasian race, diabetes mellitus, obesity, hyperparathyroidism, hyperphosphatemia, hypercalcemia, autoimmune diseases, malignancies, thrombophilic states, liver disease, hypoalbuminemia, elevated serum aluminum levels, adynamic bone disease, prolonged dialysis duration, inadequate dialysis parameters, use of calcium-based phosphate binders, warfarin therapy, and corticosteroid use, among others [1,4,6].

The pathophysiology of calciphylaxis involves dysregulation of calcium, phosphate, and parathyroid hormone metabolism, in conjunction with multiple pro-calcific factors promoting calcium deposition in the vascular wall [3,5]. Key mechanisms include osteoblastic differentiation of vascular smooth muscle cells (induced by hyperphosphatemia, uremic toxins, and oxidative stress); deficiency of physiological calcification inhibitors such as fetuin-A, osteoprotegerin, and matrix Gla protein (which is vitamin K-dependent); and activation of pro-calcific pathways, including the transcription factor NFκB, bone morphogenetic protein-4 (BMP-4), and osteopontin [4-6,8]. Additionally, a hypercoagulable state with reduced protein C and S levels, exacerbated by inflammatory cytokines (TNF-α, IL-1, IL-6), contributes to endothelial dysfunction and thrombosis [1-3,5,6,8]. The interplay of these mechanisms culminates in active vascular calcification, chronic inflammation, and microvascular thrombosis, resulting in ischemia, tissue necrosis, and ulceration [1-6,8].

Clinically, calciphylaxis typically presents with painful, non-healing skin lesions, often complicated by bullae and necrotic ulcers [2-5]. The anatomical distribution may follow a distal pattern (affecting the lower limbs), a proximal pattern (involving the abdomen, inner thighs, and gluteal region), or a mixed pattern [3,4].

Systemic involvement has also been described, with vascular calcifications affecting skeletal muscle, brain, lungs, gastrointestinal tract, optic nerve, mesentery, and male genitalia [3,4], supporting the concept of calciphylaxis as a manifestation of systemic vascular calcification [4,5].

The diagnosis relies on a high index of clinical suspicion, with skin biopsy regarded as the gold standard for confirmation [1-6,8]. Non-invasive modalities, such as bone scintigraphy, computed tomography, ultrasound, or Raman spectroscopy, require further validation and are not currently recommended for routine clinical use [1,2,5].

The differential diagnosis includes warfarin-induced skin necrosis, purpura fulminans, atheroembolic disease, antiphospholipid antibody syndrome, peripheral arterial disease, vasculitides, and necrotizing infections [1,5,6,8].

Management remains a significant clinical challenge, requiring a multidisciplinary approach involving nephrology, dermatology, plastic and reconstructive surgery, nutrition, palliative care, chronic pain management, and wound care specialists [1,5,6].

Current therapeutic strategies are largely based on retrospective studies with small cohorts, case series, and expert consensus [2,8]. Recommended interventions include pain management with opioids, correction of mineral and bone metabolism disturbances (calcium, phosphate, parathyroid hormone), specialized wound care (debridement, appropriate dressings, hyperbaric oxygen therapy), and reversal of calcification using sodium thiosulfate or vitamin K supplementation [2,8].

Prognosis depends on the pattern of involvement and early diagnosis, with sepsis being the leading cause of mortality [1,4]. Proximal involvement is associated with worse outcomes, with reported mortality rates ranging from 40% to 80% [2,3].

## Case presentation

A 71-year-old Caucasian woman has a medical history of CKD, Kidney Disease: Improving Global Outcomes G5 (KDIGO G5), secondary to diabetic nephropathy. As a relevant risk factor, she presented with secondary hyperparathyroidism related to CKD. Other comorbidities included refractory arterial hypertension, chronic anemia, and hyperuricemia.

The patient was under regular follow-up at the Nephrology Department, receiving the following medications: sevelamer 800 mg three times daily, cinacalcet 30 mg once daily, darbepoetin alfa 40 mcg twice weekly, ferrous sulfate 329.7 mg once daily, enoxaparin 20 mg once daily, febuxostat 80 mg once daily, nifedipine 30 mg twice daily, carvedilol 12.5 mg twice daily (half tablet), rilmenidine 1 mg once daily, isosorbide mononitrate 20 mg once daily, linagliptin 5 mg once daily, and omeprazole 20 mg once daily.

**Figure 1:**
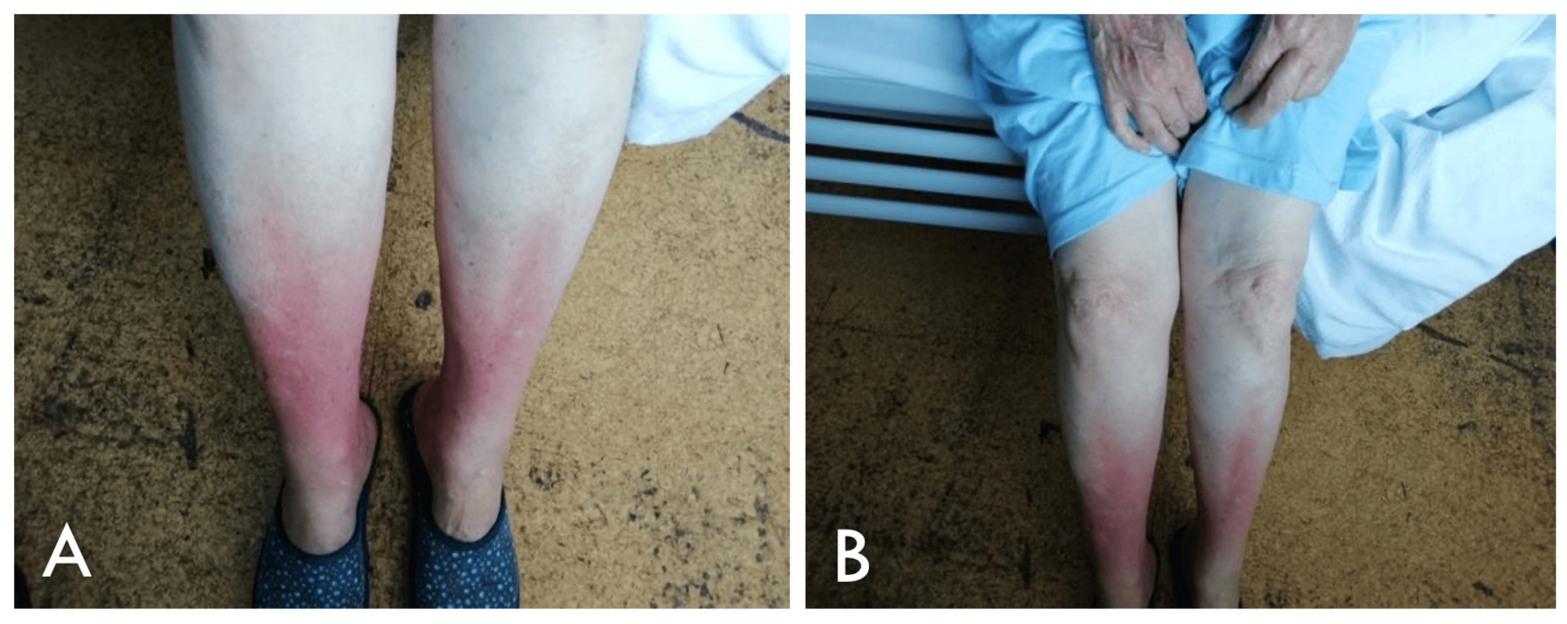
(A-B) Erythematous plaques on the lower two-thirds of the legs.

She was admitted to the Emergency Department with severe, burning-type pain in the pretibial region, with a two-day evolution. On physical examination, she presented with diffuse, poorly demarcated, grossly symmetrical, edematous, circumferential erythematous plaques on the lower two-thirds of both legs (Figures 1A-1B). Distal pulses were present and symmetrical. She was febrile (38.2 °C), with a blood pressure of 129/81 mmHg and a heart rate of 89 bpm. The remaining physical examination revealed no relevant abnormalities.

Laboratory assessment (Table 1) showed normocytic normochromic anemia (Hb: 8.7 g/dL), elevated parathyroid hormone (PTH: 885.0 pg/mL), hyperphosphatemia (6.6 mg/dL), total serum calcium of 9.0 mg/dL (corrected calcium: 10.2 mg/dL), hyperuricemia (9.6 mg/dL), hypoalbuminemia (2.5 g/dL), hypoproteinemia (5.3 g/dL), and hypokalemia (3.0 mEq/L). A 24-hour urine analysis revealed massive proteinuria (10.65 g). The remaining laboratory parameters, including coagulation studies (prothrombin time (PT), activated partial thromboplastin time (aPTT)), tumor markers (carcinoembryonic antigen (CEA), alpha-fetoprotein (AFP), CA 15.3, CA 125, CA 19.9), thyroid function, and autoantibodies, were within normal limits. Serum immunofixation showed no monoclonal component.

**Table 1:** Patient's laboratory assessment.

Parameters	Results	Reference Values
Hemoglobin	8.7	12.0-15.0 g/dL
Hemosedimentation Rate	140	<135
Creatinine	3.9	0.6-1.1 mg/dL
Ucic Acid	9.6	2.6-6.0 mg/dL
Total Proteins	5.3	6.2-8.1 g/dL
Phosphorus	6.6	2.3-4.7 mg/mL
Parathormone (PTH)	885	4.5-6.5 pg/mL
Calcium	9	8.4-10.2 mg/dL
Potassium	3	3.5-5.1 mEq/L
Albumin	2.5	3.2-4.6 g/dL

The patient was initially admitted with a presumptive diagnosis of acute lipodermatosclerosis versus fibrosing panniculitis associated with venous stasis and nephrotic syndrome.

During hospitalization, she was evaluated by a dermatologist and underwent an incisional biopsy of the posteromedial aspect of the right leg, followed by local wound care. The case was discussed with the attending nephrologist, and the decision was made to maintain the current therapeutic regimen with re-evaluation in the outpatient setting after one month.

**Figure 2:**
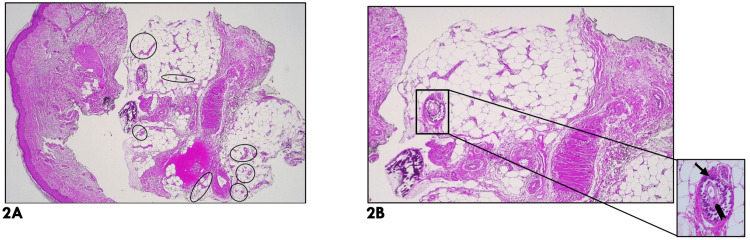
(A-B) Histopathological examination of the skin lesions. 2A (H&E, 25x) - Fragment of skin and subcutaneous tissue. Hypodermis with septal and lobular panniculitis and proliferation of small vessels arranged in a multinodular pattern (circle - indicating proliferation of small vessels). 2B (H&E, 40x) - Multiple calcium deposits are identified in the vessel walls and adipose tissue, associated with lipogranulomas. In the lower right corner (H&E, 200x), a detail of a vessel with calcified walls and a recanalized thrombus in the lumen is observed (arrow - calcifications; bracket - intravascular thrombus).

Histopathological examination of the skin biopsy (Figures 2A-2B) revealed vascular proliferation of small vessels with a multinodular arrangement. Multiple calcium deposits were identified in the vessel walls and adipose tissue, associated with lipogranulomas. These findings are consistent with calciphylaxis-associated panniculitis.

Based on clinical, laboratory, and histopathological correlation, a diagnosis of calciphylaxis was established.

Effective pain control was achieved with opioids, and topical heparinoid was initiated for cutaneous lesion management. The patient remains under regular follow-up in the Nephrology Department, with no recurrence of cutaneous ulcers to date.

## Discussion

Calciphylaxis is a rare and potentially severe disorder, still poorly understood and associated with a high mortality rate [1]. Since its initial descriptions to the present day, treatment remains challenging, requiring a multidisciplinary approach involving various specialties [1,2].

There are patterns of proximal involvement affecting the abdomen, inner thighs, and buttocks, as well as distal involvement affecting the legs, with the mixed pattern being the most common. Proximal involvement is associated with a worse prognosis and higher mortality rate [2-4]. The face and upper extremities are rarely affected [2-4]. The clinical presentation includes an initial phase characterized by tender erythematous areas, followed by the formation of reticulated livedo, plaques, and/or solitary or multiple hardened nodules. In the second phase, ulceration, necrosis, and often sepsis develop [3,5].

The patient in this case presented with distal involvement, characterized by erythematous plaques, and had risk factors such as CKD, diabetes mellitus, hyperparathyroidism, female sex, White race, hyperphosphatemia, hypoalbuminemia, and histologically confirmed calciphylaxis-induced panniculitis.

The pathophysiology is not fully understood, but it involves factors related to bone-mineral metabolism, deficiencies in inhibitors of vascular calcification (e.g., fetuin-A, osteoprotegerin, and matrix Gla protein), and other mechanisms [4,6,7]. Vascular calcification and thrombosis are necessary for the development of lesions, with the activation of the nuclear factor kB (NFkB) being a common pathway in vascular calcification [3].

Non-invasive diagnostic modalities have been proposed, but none have been systematically evaluated or are currently recommended for clinical use [5]. Bone scintigraphy may be a reliable diagnostic tool, especially when biopsy findings are inconclusive or when a biopsy cannot be performed [2]. Computed tomography, ultrasonography, and Raman spectroscopy require further investigation [1]. Differential diagnoses include warfarin-induced skin necrosis, purpura fulminans, atheroembolic disease, antiphospholipid antibody syndrome, peripheral arterial disease, vasculitis, oxalate vasculopathy, and necrotizing infections [5,6,8].

Treatment involves a multidisciplinary approach with four main goals: adequate pain control with opioids; correction of bone-mineral metabolism abnormalities in CKD, addressing calcium, phosphorus, PTH, and calcium-phosphorus product levels; optimization of wound care with judicious use of surgical debridement, dressings, and hyperbaric oxygen therapy; and halting the progression and reversing vascular calcification with sodium thiosulfate (STS) or vitamin K supplementation [2,8]. The treatment of the patient in our case involved multiple specialties, including dermatology, nephrology, and internal medicine.

Current treatment recommendations lack robust data, as most evidence comes from retrospective studies with small samples, case series, and expert opinions [2,3].

This clinical case aims to emphasize the importance of disseminating research on this topic and to promote greater awareness among clinicians regarding the need for early diagnosis and treatment, with the goal of preventing cutaneous necrosis.

Serum calcium and phosphorus levels should be maintained within normal ranges, and calcium supplements or calcium-based phosphate binders should be discontinued [7]. Sevelamer, at a dose of 800 mg three times daily, is recommended for controlling hyperphosphatemia with a lower risk of extraosseous calcifications [2,7].

Intact PTH levels should be maintained between 150 and 300 ng/mL. Cinacalcet, at a dose of 30-60 mg once daily, has been shown to reduce PTH levels to the desired range [4,7].

Intensifying the dialysis regimen may help maintain normal serum calcium and phosphorus levels, thereby reducing the incidence of calciphylaxis, limiting lesion progression, and promoting faster healing [2,5]. Bisphosphonates have been used successfully to effectively reduce calcium-phosphorus levels [5]. The role of parathyroidectomy in treating calciphylaxis remains controversial [2,3].

STS is considered by many as first-line therapy, significantly contributing to the treatment of calciphylaxis due to its calcium-chelating, vasodilatory, and antioxidant properties [2]. The dose is 12.5-25 g intravenously during the last hour of hemodialysis sessions [2-4]. Treatment duration varies from three to six months or until complete wound healing. Pain relief and reduction in skin lesions are typically observed after a few weeks of STS use [2-4].

Direct wound management includes debridement, regular dressing changes, and hyperbaric oxygen therapy [2,4,7]. Surgical debridement, in selected cases, has been associated with survival benefits in some small retrospective studies [2].

Hyperbaric oxygen therapy has been proposed as a second-line treatment for wound healing, with good results in retrospective studies, but claustrophobia, access, and cost may limit its use [2,5].

For adequate pain control, fentanyl and methadone are preferred over morphine, as they lack active metabolites that accumulate in renal failure [2,5,6]. The use of nonsteroidal anti-inflammatory drugs (NSAIDs) is limited in these patients [2,5]. Given the severity and complexity of pain, palliative care teams can play a crucial role in managing calciphylaxis [2,5].

In small case series, maggot therapy, kidney transplantation, low-density lipoprotein (LDL) apheresis, and vitamin K supplementation have been used in the treatment of calciphylaxis [8]. Alternative approaches still lack robust evidence from clinical studies to be recommended as a treatment for widespread use [2,8].

## Conclusions

This complication presents with a wide spectrum of clinical manifestations, ranging from changes in skin coloration to deep necrotic ulcers. The possibility of occurrence in individuals with preserved renal function is also noteworthy.

Given this clinical variability and the potential severity of the condition, early diagnosis is imperative to prevent progression to sepsis, which is the leading cause of mortality.
